# Penile Carcinoma: Risk Factors and Molecular Alterations

**DOI:** 10.1100/tsw.2011.24

**Published:** 2011-02-03

**Authors:** Marilia F. Calmon, Mânlio Tasso Mota, José Vassallo, Paula Rahal

**Affiliations:** ^1^ Departamento de Biologia, Instituto de Biociências, Letras e Ciências Exatas, São José do Rio Preto, São Paulo, Brazil; ^2^ Hospital A. C. Camargo, São Paulo, Brazil

**Keywords:** penile, carcinoma, risk factors, molecular alterations

## Abstract

Penile carcinoma is a rare, male cancer. Although the incidence of penile carcinoma is very low in Western countries, in some countries, the incidence is significantly greater, with penile carcinoma accounting for ≤10% of all male malignancies. Greater insight has been gained in recent years as to its pathogenesis, the risk factors associated with its development, and the clinical and histological precursor lesions related to this disease. In this review, risk and conditions factors for penile carcinoma, molecular alterations in this type of cancer, histological types, and prognostic factors will be discussed in order to further our understanding of the biology and behavior of this cancer.

## INTRODUCTION

The incidence rate of penile carcinoma varies enormously among different populations; some developing countries are most seriously affected. The disease can reach as high as a 10% incidence rate in men in some African, Asian, and South American countries (4.2 and 4.4/100,000 in Paraguay and Uganda, respectively[1,2]). This tumor represents 2% of all cancer cases in Brazilian men, being more frequent in the North and Northeast regions than in the South and Southeast regions. In the regions of high incidence, the number of cases is higher than those of prostate and bladder cancer[3]. In Western Europe and the U.S., age-standardized incidence rates range from 0.3 to 1.0/100,000, accounting for 0.4–0.6% of all malignancies in these parts of the world[4]. The substantial worldwide variation in penile carcinoma incidences may be linked to differences in socioeconomic and religious conditions[5].

Although squamous cell carcinoma (SCC) of the penis can occur at any age[6], most cases affect individuals of advanced age, with a peak of incidence ~60 years old[7,8]. Familial occurrence has rarely been documented[9].

Penile carcinomas usually have a squamous epithelial origin and include carcinomas *in situ*. The latter are known as erythroplasia de Queyrat and Bowen disease. These diseases have similar histological appearance and biological behavior and, therefore, are frequently considered as different aspects of a single preneoplasic disorder[10,11]. Invasive carcinoma is represented by SCC and its variant of low-grade well-differentiated verrucous carcinoma, frequently reported as giant condyloma acuminatum or Buschke-Loewenstein tumor, although the classification of these diseases remains controversial.

Therefore, the best classification is giant condyloma acuminatum or Buschke-Loewenstein tumor as verrucous carcinomas of the anogenital mucosae[12,13].

The clinical presentation of penile carcinoma varies from a subtle swelling; a small lump, pustule, or warty growth; to an extensive carcinoma with peeling. The earlier symptoms of penile carcinoma include itching or burning under the foreskin of the glans or ulceration of the foreskin. The carcinoma originates mainly in the glans (80%), followed by the prepuce (15%), the balanopreputial membrane (5%), and more rarely in the shaft. Macroscopically, it may present as vegetative or ulcerated lesions. The vegetative lesions have a better prognosis because they tend to grow out, whereas ulcerated lesions are more infiltrative and metastasize more frequently[14].

The progression of penile carcinoma occurs in a locoregional manner, with sequential involvement of the sentinel (inguinal and pelvic) lymph nodes before the development of distant metastasis. The common sites of metastases include lung, brain, and bone. Tumor recurrence may develop in the lymphatic system with discrete or distant nodes. Death often occurs as a result of starvation and sepsis or bleeding as a result of erosion caused by femoral venous ulceration of inguinal metastatic involvement[14].

This review looks primarily at the risk and conditions factors for penile carcinoma, as well as the possible molecular alterations that lead to histologically distinct tumor, and into prognostic factors to get a better understanding of its biology and behavior.

## METHODOLOGY

A Medline search using PubMed with the search terms “penile/penis cancer/carcinoma” revealed 5004 published papers between 1956 and 2010. A manual search for publications with strictly direct relevance to the risk factors, the molecular and genetic changes, and potential HPV involved in the molecular genetics of its etiology resulted in 80 papers being identified. These form the basis of the following review, being augmented by other articles on the epidemiology and management of penile carcinoma where appropriate.

## RISK CONDITIONS

The etiology of penile carcinoma seems to be multifactorial, but some risk conditions and risk factors have been identified. Penile tear, rash (defined as chronic dermatitis lasting >1 month), injury, and inflammation were associated with an increased risk for cancer development[15]. Some studies have also associated penile carcinoma with inflammation together with injury, as many penile carcinomas arise at sites of infection, chronic irritation, or injury[16,17].

Another risk condition in the development of SCC is PUVA therapy (photochemotherapy using psoralen and ultraviolet [UV] A irradiation) used in the treatment of skin diseases such as psoriasis, vitiligo, and cutaneous T-cell lymphoma[18,19]. Injection of mineral oil into the skin of the corpus cavernosum region can also be a risk condition. This injection may cause an inflammatory reaction, confirmed by the presence of lipogranulomas and foreign body reactions[20].

Hispanics have the highest rates of primary malignant penile carcinoma compared to any other racial/ethnic group, followed by Alaskan Native/American Indians, Africans, Caucasians, and non-Hispanics[21]. It is possible that these racial/ethnic effects are due to differences in sexual and other behavior patterns that may lead to HPV exposure. Because genital HPV infection is spread by sexual contact, behavioral differences between racial and ethnic groups could partially explain these variations in rates. Indeed, high school students of Asian-Pacific ancestry are significantly less likely to have ever engaged in sexual intercourse than African, Caucasian, or Hispanic students[5,22].

Madsen et al.[23] suggest that the transmission of etiological factors responsible for penile carcinogenesis, presumably HPV16 and others hrHPVs, frequently occur by intimate contact. There is a significant trend of increased risk with an increase in the number of female sexual partners. Notably, the number of partners of <20 years of age seems to differ between the patients diagnosed with SCC of the penis and the controls. This study showed a strong and significant association between oral-penis sex and the risk of SCC, suggesting that oral sex, a common sexual practice in almost half of the control population in Denmark, may have an underestimated role in viral transmission from the oral cavity/pharynx of the female partner to the penis.

## RISK FACTORS

### Use of Tobacco

Although an association with smoking has been repeatedly observed, the exact role that smoking plays in the development of this disease remains unknown. There is an association between men who smoked when they were diagnosed with penile carcinoma, both *in situ* and invasive[15,17]. Hellberg et al. found a clear dose-response relation, with smokers of >10 cigarettes/day having a significantly greater risk than light smokers[24]. Maden et al. found that the risk of penile carcinoma among men who smoked at diagnosis was 2.8 times that of men who had never smoked, and lifetime smoking of >45 pack-years of cigarettes elevated the risk to 3.2 times that of men who had never smoked[16]. Tobacco might act through its metabolites or directly after systemic absorption[25]. Smoking seems to have an important role in cases where cigarette smokers had been diagnosed with penile carcinoma, although it may be more important in the advanced stages of progression[15].

### Human Papillomavirus (HPV)

The HPV is a family of small, double-stranded DNA viruses (8000 bp) of distinct pathogenetic types. There are >100 known variants of HPV. Muñoz et al. have classified HPV types according to their oncogenic potential (Table 1)[26]. Sexual transmission is the most common route for viral propagation, although both oral and vertical transmission have been described[27].

**TABLE 1**
**HPV Types and Oncogenic Potential**

| Classification | HPV Types |
|---|---|
| High risk | 16, 18, 31, 33, 35, 39, 45, 51, 52, 56, 58, 59 |
| Probably oncogenic | 26, 53, 66, 68, 73, 82 |
| Low risk | 6, 11, 40, 42, 43, 44, 54, 61, 70, 72, 81 |

Modified from Muñoz et al.[26].

Positivity for HPV DNA is strongly associated histopathologically with the basaloid/warty subtypes and is weakly associated with keratinizing/other SCCs. There is only a weak or no association between verrucous carcinoma of the penis and HPV positivity, but this depends on the origin of the population under review[2,28,29,30].

The common HPV types are HPV16 and HPV18. HPV16 is more prevalent in North America, Europe, South America, and India[30,31]. Although HPV18 is the second type more usually identified in the samples of SCC, it has a low prevalence among all the histological types. HPV infection may influence prognosis in patients with penile carcinoma. Traditionally, high-risk HPV types, especially with

HPV16 and 18, were thought to be associated with aggressive variants and, therefore, to give rise to poorer survival than non-HPV–associated penile carcinoma.

Penile carcinoma is a heterogeneous disease and a proportion of these carcinomas can be attributed to HPV infection, whereas a number of other molecular mechanisms causing penile carcinoma independently of HPV probably represent the most common events. However, HPV16 remains an important etiologic factor[15,24].

Cancer of the penis associated with HPV appears from a progression of precursor lesions caused by the viral infection. Some researchers believe that the pathway is similar to carcinogenesis of the uterine cervix mediated by HPV. Carcinogenesis induced by HPV involves several steps: resistant infection caused by HPV is the initial causal event, with genetic[32] and epigenetic alterations[33,34,35,36]. It is necessary for a HPV-infected cell to acquire a malignant phenotype.

The HPVs exert their oncogenic effects through expression of the E6 and E7 oncoproteins, which bind and inactivate p53 and pRB proteins, products of tumor-suppressor genes[37,38]. Their expression is needed to induce and maintain the neoplastic phenotype of cervical cancer cells, and similar mechanisms may therefor apply in penile carcinoma. By means of imbalance of the $p14^{ARF}$/MDM2/p53 and $p16^{INK4A}$ pathways, the oncogenic HPVs interfere with cell cycle control, affecting both proliferation and apoptosis. Functional disruption of pRB by E7 of the HPV results in the reciprocal overexpression of $p16^{INK4A}$, due to a negative feedback between pRB and $p16^{INK4A}$[39,40]. The genetic and epigenetic events subsequent to the cell-host interaction remain poorly understood.

Until today, some studies identified nonviral mechanisms that can lead to an imbalance of both the $p14^{ARF}$/MDM2/p53 and $p16^{INK4A}$/cyclin D1/RB pathways in a subset of penile carcinoma. There are at least two possible mechanisms that can disrupt the latter pathway during carcinogenesis in the absence of HPV. One mechanism is the silencing of the $p16^{INK4A}$ gene through hypermethylation of its promoter region. Another mechanism is the overexpression of the gene BMI-1 from the polycomb group, by which the $p16^{INK4A}$ pathway may be disturbed[41]. The INK4A/ARF locus, encoding both $p16^{INK4A}$ and $p14^{ARF}$, was identified as a critical downstream target of BMI-1. In mice, BMI-1 overexpression results in decreased expression of the respective genes.

## Human Immunodeficiency Virus (HIV)

Sexual transmission of HIV requires the virus to penetrate epithelial tissue. The inner lining of the prepuce provides such an access route. This is because it is a mucosal epithelium with a protective keratin layer that is very much thinner than in the outer prepuce and glans penis[42]. The uncircumcised penis is more susceptible to minor trauma and ulcerative disease, and the preputial sac serves as a reservoir for pathogenic organisms present in the pool of smegma (a whitish film consisting of neutral lipids, fatty acids, sterols, and exfoliated cells) that accumulates beneath the prepuce. Because of the potential for infection these pose, the mucosal epithelium has a high prevalence of immune system cells, including CD4+ T cells, Langerhans cells, and macrophages.

The mechanism by which the immunosuppression related to HIV plays a role in the increased risk of cancer associated to HPV is not yet clear. Among the individuals infected with HIV, the incidence of invasive cancers associated to HPV does not seem to have a relationship with CD4-T-cell count, used to measure the immune pattern[43,44]. Comprehension of associations of immunosuppression and an active antiretroviral therapy (HAART) used against the HIV virus with the risk of cancer associated to HPV is important because the recovery of the immune system from HAART is partial. Prolonged survival of individuals infected with HIV along with a compromised immune system could lead to an increased incidence of cancers weakly related to immunosuppression[44,45].

Chaturvedi et al.[46] observed that there is a higher risk of patients infected with HIV developing penile carcinoma compared to the general population in the U.S. A higher risk of developing penile carcinoma *in situ* is found in patients infected with HIV than invasive cancer associated to HPV. Studies reported a higher incidence of penile carcinoma in individuals infected with HIV in the U.S. and Uganda[47,48].

## Smegma

Improved penile hygiene is the main reason for circumcision. Smegma has been cited by men as dirty and infected with micro-organisms. Improved penile hygiene frequently is difficult to achieve, and sometimes the attempts to get a good asepsis can cause dermatologic problems in uncircumcised men. To the parents, it is easier to keep their childrens' penises clean when they have been circumcised[49]. There is a trend in circumcised men to take showers and retract the skin in order to clean the penis more frequently than in uncircumcised men[50].

Production of the smegma increases in younger people, peaking between 20 and 40 years of age. Initially, smegma is a yellow or white lubricant. In time, it transforms chemically from a lubricant to a mixture of epithelial cells, dirt, and micro-organisms that together form aggregates and generate an offensive odor. For a long time, it was believed that smegma could be carcinogenic[51]. Some studies between 1940 and 1960 tried to find such evidence[52,53,54], some of which are still considered valid by several researchers. However, these studies were based in animal models, in which human smegma was introduced into skin tunnels or the vagina of mice. The evidence of association with cancer development is weak and could be explained by the chronic inflammation due to an exogenous antigen in the mucosa. Therefore, this association does not seem to be based on scientific evidence[49].

## Phimosis/Circumcision

Phimosis is pathology characterized by the inability to retract the prepuce to expose the gland; it occurs in uncircumcised men or men who were inappropriately circumcised. Several studies have associated the history of phimosis with an increase in risk of the development of penile carcinoma[23,24]. Phimosis is also linked to others pathologies, such lichen sclerosus and giant condyloma (Buschke-Löwenstein tumor)[55]. However, circumcision seems to decrease the risk of penile carcinoma only when it is performed in childhood.

Neonatal circumcision has been well established as an effective prophylactic measure for penile carcinoma. Its protective effect is mainly explained by the fact that certain conditions, such as phimosis, related to the retention of smegma and lichen sclerosus are less prevalent in neonatal circumcised men[56]. Some reseachers observed that men who were not circumcised in childhood have a higher risk of invasive penile carcinoma and phimosis compared to circumcised men[15,57]. On the other hand, Madsen et al.[23] observed that there is no difference in the risk of penile carcinoma between men circumcised in childhood and uncircumcised men.

Apparently, the increase in the risk of penile carcinoma in men who were not circumcised in childhood is related to the increase in the risk of phimosis. This indicates that the risk of penile carcinoma can be reduced by preventing the development of phimosis through circumcision in childhood. In uncircumcised men, phimosis can cause injuries that can facilitate access of HPV to the basal epithelium. Also, phimosis can cause inflammation of the glans or prepuce that may result in chronic infection, which increases the risk of penile carcinoma even with only a few sexual partners[15]. Besides, circumcision may protect against HPV-associated disease by enhancing the resolution of infection[58].

## Lichen Sclerosus (LS)/Balanitis Xerotica Obliterans (BXO)

Penile LS was first described in 1928 by Stuhmer[59]. Most authors consider LS as synonymous with BXO. It is a chronic, sclerotic, inflammatory disease leading to phimosis and meatal stenosis. The lesions are initially polygonal, flat-topped plaques of white color that progress to atrophic and sclerotic plaques. The most commonly affected anatomical sites are the glans and foreskin, but other regions (such as the frenulum, urethral meatus, and anterior urethra) may also be involved. Less commonly, LS can involve the shaft of the penis. Hemorrhage is common when there is glans involvement. Additionally, hemorrhagic bullae, erosions, and fissures may be present. A typical finding is a sclerotic white ring at the tip of the prepuce[55].

The process is benign, but the lesions lead to mucosal contraction and subsequent phimosis with progression of the disease. In advanced cases, meatal stenosis can cause urinary flow obstruction and requires surgical treatment[60]. Symptoms are pruritus, burning, priapism, difficulty in retracting the foreskin, dysuria, and a poor urinary stream. SCC of the penis arising on a background of LS manifests between 36 and 83 years of age, with a mean of 54[61], being more common in uncircumcised men[55]. The etiopathology of LS remains unknown, but some possible causes are autoimmunity, infection (*Borrelia burgdorferi*, HPV, hepatitis C virus [HCV]), hormonal factors, traumas (burnings, radiotherapy, surgery), and meatal stenosis[62].

The association between LS and SCC in women is widely recognized. The risk of malignant transformation with vulvar LS is 3–6%[55]. LS, SCC, and HPV are more common in uncircumcised men, but the association between LS and SCC in men is unproven. The available data in literature seem contradictory. In retrospective studies with patients who progress to SCC, high rates of LS are found. In a study of 20 patients bearing SCC, 50% showed evidence of LS[61]. In another study, the clinicopathologic data of 207 SCC and giant condyloma samples were verified and 68 (32.9%) patients with LS were identified[56]. In a group of 18 patients with SCC, Perceau et al.[63] found eight (44%) with LS.

However, in studies of patients with LS who progress to SCC, only a small percentage led to some kind of malignant transformation. In a review of 130 LS cases, Barbagli et al.[64] found 11 (8.4%) patients who developed malignant or premalignant alterations. In a study of 54 patients with LS, only two (3.7%) cases of tumor were noted[65]. Nasca et al.[66] found malignant alterations in only six of 86 (5.8%) patients with LS; in another study, the same group found eight (9.3%) patients who developed epithelial carcinoma in a cohort of 86 patients[67].

Some studies have demonstrated a wide range in lag time from LS diagnosis to cancer development[66]. The data taken together suggest that LS must act as a catalyst and not as a primary carcinogenic, since the development of SCC from LS is unusual. Perhaps the development of phimosis predisposes the tissue by accumulation of smegma or even allows HPV viability for longer periods[68].

## INGUINAL METASTASIS

The survival of the patients bearing penile SCC is associated with the presence and extent of inguinal node metastasis[69]. The presence of metastatic lymph nodes was the best prognostic factor for patient survival found in a study of 202 cases of penile SCC. The rate of inguinal/regional metastatic lymph node involvement in patients with clinical stages N0, N1, and N2 were 24, 70, and 42%, respectively. Patients with negative lymphadenectomy had better survival rates at 10 years than those with positive lymphadenectomy[70]. In another study of 333 cases of penile SCC, half the patients who underwent total or partial penectomy showed inguinal lymph node metastasis. In contrast, patients who underwent circumcision or local excision did not show iguinal lymph node metastasis and had a cure rate of 100%[71].

## HISTOLOGICAL TYPES

SCC accounts for 95% of all penile neoplasias. It is classified in the subtypes: usual type, verrucous carcinoma, warty carcinoma, papillary carcinoma, basaloid carcinoma, sarcomatoid carcinoma, carcinoma cuniculatum, pseudohyperplastic carcinoma, adenosquamous carcinoma, and acantholytic carcinoma[72]. Major histological types are given in Table 2.

**TABLE 2**
**Characterization of Penile SCC Histologic Types that have Higher Distributions**

| Carcinoma Subtype | Distribution (%) | Main Features | Metastatic Rate (%) | Prognosis |
|---|---|---|---|---|
| Usual | 48–65 | Most common subtype, morphologically similar to SCC from other sites | 28–39 | Intermediate |
| Basaloid | 4–10 | HPV-related aggressive tumor composed of deeply invasive tumor nests of basaloid cells | 50–100 | Ominous |
| Verrucous | 3–8 | Low-grade verruciform tumor, usually invading only superficial anatomic levels | Null | Excellent |
| Warty | 7–10 | HPV-related verruciform tumor similar to warty carcinomas from other sites | 17–18 | Good |
| Papillary | 5–15 | Low-grade verruciform tumor frequently invading superficial erectile tissues | 12 | Good |

Modified from Chaux et al.[72].

SCC can also be divided on morphological grounds into two groups: exophytic (fungating, papillary) and endophytic (infiltrative, ulcerating). The exophytic form is well differentiated and heavily keratinized, and may grow into a large polypoid mass. Exophytic penile carcinoma is commonly located in the glans and may occur in the prepuce. The endophytic form is more undifferentiated, occurring in the foreskin, glans, and more rarely in shaft. Ulcerating tumors have higher rates of lymph node metastasis[73]. Cubilla et al.[74] classified SCC of the penis into five categories: superficial spreading, vertical growth, verruciform, multicentric, and mixed. The superficial growth tumors are the most frequent and inguinal metastasis is present in 42% of the cases.

Metastatic lymph nodes were found in 82% of the deep vertical growth tumors, 33% of the multicentric cases, and none with verrucous pattern. The verrucous carcinoma is responsible for 5% of all penile carcinomas. The tumor can be locally aggressive, but it is biologically benign and, if correctly disgnosed, shows no metastasis[71]. Microscopically, it is an extremely well-differentiated papillary SCC, with the same characteristics of the homonym tumor of the superior aerodigestive tract[73].

Nonsquamous primary malignancies make up <5% of the penile carcinomas. Among them, the sarcomas (including Kaposi's sarcoma) are the more frequent, followed by melanomas, basal cell carcinomas, extramammary Paget's disease, and lymphomas. Carcinomas rarely metastasize to the penis. Priapism is one form of presentation. The most common sources are, in decreasing order of frequency, prostate, bladder, rectosigmoid colon, kidney, and testis.

## MOLECULAR ALTERATIONS

The rarity of penile carcinoma, together with the technical difficulties inherent in this tumor because of the presence of inflammation, necrosis, and poor growth in cell culture, have led to few results and reviews about molecular alterations of penile carcinoma[75,76]. Table 3 summarizes the molecular alterations observed in penile carcinoma.

There are alterations in the number of the DNA copies in samples of penile SCC, with similarities with other types of SCCs, such as oral and esophageal carcinoma. There is gain in the number of DNA copies, including 8q24, 16p11-12, 20q11-13, 22q, 19q13, and 5p15, and deletions in 13q21-22, 4q21-32, and along the X chromosome[32]. Ornellas et al. and Masih et al.[77,78] discovered a diploid population in verrucous carcinomas. Ornellas et al. also observed that aneuploidy varied according to grade of the penile SCC. Unfortunately, there were only three poorly differentiated tumors in this study, preventing

**TABLE 3**
**Molecular Alterations Observed in Penile Carcinoma**

| Molecular Alterations | Gene/Chromosome | Refs. |
|---|---|---|
| Copy number gains | 8q24, 16p11-12, 20q11-13, 22q, 19q13, and 5p15 | [32] |
| Deletions | 13q21-22, 4q21-32 and along the X chromosome | [32] |
| Aneuploidy | Aneuploidy in invasive penile SCC | [77] |
| Gene overexpression in HPV-positive patients | *p53* | [83] |
| Gene overexpression | *p53, p21, RB,* and *NF-κB* | [87,88] |
| Gene low expression | *p16* $^{INK4a}$ | [87] |
| Gene mutations | *p53, c-ras* $^{Ha}$ | [89] |
| | *PIK3CA, HRAS,* and *KRAS* | [92] |
| Imbalance protein expression | Bcl-2/Bax | [93,94] |
| DNA hypermethylation in the promoter region of the gene | *DAPK, FHIT, MGMT, p16* $^{INK4a}$, *p14* $^{ARF}$, *RAR-β, RASSF1A, RUNX3,* and *TSP-1* | [33,34,35] |

formal analysis of aneuploidy as a prognostic factor[77]. Nonetheless, there was a tendency towards a high DNA index correlating with increased metastatic risk.

Several studies have investigated the relation between *p53* expression and HPV infection, as well as the relation of *p53* expression to prognosis of penile carcinoma[2,79,80,81]. The HPV oncogenic product, E6, is known to interfere with this pathway by binding to the oligomerization region of wild-type *p53* to mediate its ubiquitination and degradation by 26S proteasome via a pathway that is thought to be analogous to MDM2-mediated p53 degradation. Binding of E6 to p53 causes suppression of normal *p53* inhibitory function at the G1/S transition of the cell cycle, causing uncontrolled cell proliferation, loss of differentiation, and decreased apoptotic control through BCL-2 and BCL-2–associated X protein (BAX) imbalance. Blanton et al.[82] produced an *in vitro* model in which foreskin and ectocervical epithelial cells were infected with retroviral vectors expressing HPV16 oncogenes. Expression of *E7* caused persistent proliferation in the suprabasal domain, but no effect on terminal differentiation. Cells expressing only *E7* were positive for *p53*, whereas cells coexpressing *E6* and *E7* were negative, but still proliferating suprabasally. The authors concluded that *E7*-induced suprabasal proliferation is independent of the amount of baseline *p53*.

The evidence linking *p53* expression and presence of HPV DNA in penile carcinoma is contradictory. Lam et al.[83] reported that 100% of HPV-infected cells also showed positive p53 staining. However, several reports showed an inverse or negative relationship between these two factors, reviewed the expression profile of premalignant conditions (treatment-resistant genital warts, bowenoid papulosis, erythroplasia de Queyrat, and carcinoma *in situ*), and compared this with the profiles of invasive tumors. They concluded that there was no correlation between *p53* expression and HPV status. Overexpression of *p53* does not indicate a *p53* mutation in premalignant disease, which infers that *p53* gene mutations might occur in the later events of male genital carcinogenesis and, thus, describe a subset of patients with alternate disease progression[84,85,86].

Lam et al.[83] developed their initial findings of p53 expression in penile carcinoma to include p21 expression data, so that the interplay between these two proteins could be investigated. Nuclear staining for the p21 protein was found in 40% of tumors, with a suprabasal localization. This staining was also seen in adjacent dysplastic and carcinoma *in situ* areas with no correlation to grade or stage. There was a strong association between HPV16 positivity and positive p21 immunostaining (100%). The authors suggest that, in penile carcinoma, p21 expression is controlled by p53-dependent and p53-independent mechanisms. Stankiewicz et al.[87] failed to observe a significant association between p21 protein expression and histological types of penile carcinoma.

The $p16^{INK4a}$/cyclin D/retinoblastoma pathway is of specific interest in the pathogenesis of penile carcinoma. This pathway is deregulated in several types of human carcinomas, with inactivation of single or multiple steps within the pathway. Retinoblastoma is a target for the viral oncoprotein E7; thus, its inactivation might contribute to carcinogenic events in HPV-dependent tumors.

Ferreux et al.[41] showed that this pathway can be disrupted by three independent mechanisms in penile carcinoma. They described HPV-dependent and HPV-independent mechanisms affecting the normal functioning of this important signaling pathway. These mechanisms included blocking of retinoblastoma function by the E7 oncoprotein with up-regulation of $p16^{INK4a}$ and silencing of the $p16^{INK4a}$ gene by methylation in the absence of HPV infection, with overexpression of $p16^{INK4a}$ in 15% of cases. Overexpression of *BMI-1*, which targets the downstream factors $p16^{INK4a}$ and $p14^{ARF}$, was also noted in 10% of cases in the absence of HPV infection. The authors postulated that $p16^{INK4a}$ might act as a potential prognostic marker and that these results have strong implications on the potential effectiveness of prophylactic HPV vaccines for this tumor[41].

An immunohistochemical analysis of several proteins associated to cell cycle and HPV detection in penile carcinoma samples has been carried out in which it was observed that $p16^{INK4a}$ and Ki-67 expression were significantly lower in verrucous carcinoma than in the common type of SCC, suggesting that low levels of $p16^{INK4a}$ and Ki-67 expression differentiate a verrucous penile carcinoma from a common type of SCC. Besides, HPV infection was detected in only 23% of the samples analyzed. The low level of $p16^{INK4a}$ expression and HPV detection suggests that the pathogenesis of penile verrucous carcinoma is unrelated to HPV infection, neither oncogenes nor suppressor tumors genes classically altered by viral infection[87]. On the other hand, another study of penile carcinoma samples from Kenya failed to find any difference in $p16^{INK4a}$ expression between penile carcinomas that were HPV negative or positive[88].

Proto-oncogene activity in penile carcinoma has been analyzed through $c\text{-}ras^{Ha}$ and *myc* mutations and their relation with HPV involvement. Leis et al.[89] assessed two patients with penile carcinoma, with one developing late relapse from inguinal metastasis in the seventh year. HPV18 was isolated in the primary tumor and nodal metastasis, which was linked to mutations in *p53* and $c\text{-}ras^{Ha}$. Although HPV infection can be involved in early carcinogenesis, mutations in *ras* genes occur later and are linked to disease progression. Couturier et al.[90] and Sastre-Garau et al.[91] investigated the pattern of genomic integration of HPV DNA in a small number of patients with penile carcinoma. These studies highlighted the fact that the integration of HPV types 16 and 18 was localized to sites containing *c-myc* (8q24.1) and *n-myc* (2p24) proto-oncogenes. Another study analyzed possible mutations in *PIK3CA, PTEN, BRAF, HRAS, KRAS,* and *NRAS,* and observed that *PIK3CA* mutations were found in all grades and stages, whereas *HRAS* and *KRAS* mutations were present in larger and more advanced tumors[92].

The complex interaction between the proapoptotic protein BAX, the antiapoptotic BCL-2, and the p53 deregulation is well known in several tumors. Saeed et al.[93] found no difference in BAX expression between the verrucous carcinoma and well-differentiated SCC. Nascimento et al.[94] and Saeed et al.[93] reviewed this area in penile carcinoma. Both studies found an imbalance in BAX/BCL-2 ratios between benign, premalignant, and invasive disease. BCL-2 concentrations were significantly increased in low-grade disease compared with verrucous cancers, whereas BAX concentrations were comparable. The average proportion of BCL-2/BAX was significantly low in verrucous carcinoma compared to well-differentiated SCC, and the proportion of BCL-2/BAX was considered one of the strongest independent predictors of disease progression and prognosis[95,96]. High proportions of BCL-2/BAX in poorly differentiated squamous tumors and an increase in the proportion of BCL-2/BAX are associated to poor outcomes in others types of tumors. Nascimento et al.[94] observed an imbalance in the proportions of BCL-2/BAX between the benign, premalignant, and invasive lesions, suggesting that the imbalance of BCL-2 and BAX is probably associated with the neoplasic process.

Epigenetic mechanisms involving DNA methylation, histone modifications, and noncoding RNAs regulate and maintain gene expression states. Similar to genetic mutations, alterations in epigenetic regulation can lead to uncontrolled cell division; tumor initiation; and growth, invasiveness, and metastasis[97].

The role of DNA methylation in cancer has received much attention; it is accepted that the methylation of the promoter region of many genes is associated with gene silencing[98]. DNA methylation is the addition of a methyl group for the carbon atom 5'-cytosine, present in the dinucleotide CpG, resulting in the formation of a 5-methylcytosine[99]. The methyl groups found mainly on the islands of the CpG promoter regions of genes can influence the expression of DNA into proteins, reducing the binding affinity between the promoter regions and transcription factors through mechanisms involving changes in chromatin structure or levels of histone acetylation[100].

Yanagawa et al.[35] analyzed the methylation pattern of several genes in penile carcinoma samples and observed hypermethylation in the genes *DAPK*, *FHIT*, *MGMT*, $p14^{ARF}$, $p16^{INK4a}$, *RAR-β*, *RASSF1A*, and *RUNX3*, suggesting that in the absence of HPV, pathogenesis of SCC may require alterations of a number of genes, including *p53* gene mutations and methylation of suppressor tumor genes, or genes related to tumor than the pathogenesis with HPV infection[35]. Guerrero et al. observed hypermethylation in the genes *TSP-1*, *RASSF-1*, and $\text{p16}^{\text{INK4a}}$ in penile SCCs, and the hypermethylation of the gene *TSP-1* was significantly associated with unfavorable histological grade and vascular invasion[33]. Another study analyzed the methylation status of several genes and found that at least one of the genes was hypermethylated in all the samples analyzed. With the exception of the gene *FHIT*, the genes *DAPK*, *MGMT*, $p16^{INK4a}$, *RAR-β*, and *RUNX3* also were hypermethylated in >20% of the samples, suggesting that the methylation of the suppressor tumor genes or genes related to tumor affects the pathogenesis of penile carcinoma as well as others carcinomas[34].

## CONCLUSION

Penile carcinoma is a severe, uncommon disease mainly related to poor hygiene, sexual history, and even smoking. Developments in the understanding of the pathogenetic changes that occur in penile carcinoma have been limited, mostly because of the small numbers and paucity of pathological tissue for preclinical research. The comparative rate of progress to other tumor types is fundamentally a result of the rarity of this disease, and only a small number of investigations have focused on the molecular and genetic alterations. This review shows the risk factors related to penile carcinoma, and limited data of the molecular and genetic alterations related to the pathogenesis of this type of cancer. It is essential to develop strategies to inform the population of developing countries, mainly the Brazilian population, about the importance of personal hygiene for men, the risk factors involved in penile carcinoma, and the importance of early diagnosis of this type of cancer in order to improve both mortality and management strategies.
